# The *Sinorhizobium meliloti* NspS-MbaA system affects biofilm formation, exopolysaccharide production and motility in response to specific polyamines

**DOI:** 10.1099/mic.0.001293

**Published:** 2023-01-30

**Authors:** Víctor M. Chávez-Jacobo, Víctor A. Becerra-Rivera, Gabriela Guerrero, Michael F. Dunn

**Affiliations:** ^1^​ Programa en Genómica Funcional de Procariotes, Universidad Nacional Autónoma de México, Cuernavaca, Morelos, 62210, Mexico; ^2^​ Unidad de Análisis Bioinformáticos, Centro de Ciencias Genómicas, Universidad Nacional Autónoma de México, Cuernavaca, Morelos, 62210, Mexico

**Keywords:** biofilm, cyclic di-GMP, exopolysaccharides, motility, NspS-MbaA, polyamine sensing, *S. meliloti*

## Abstract

We previously showed that specific polyamines (PAs) present in the extracellular environment markedly affect extracellular polysaccharide (EPS) production, biofilm formation and motility in *Sinorhizobium meliloti* Rm8530. We hypothesized that extracellular PA signals were sensed and transduced by the NspS and MbaA proteins, respectively, which are homologs of the PA-sensing, c-di-GMP modulating NspS-MbaA proteins described in *Vibrio cholerae *. Here we show that the decrease in biofilm formation and EPS production in the quorum-sensing (QS)-deficient *S. meliloti* wild-type strain 1021 in cultures containing putrescine or spermine did not occur in a 1021 *nspS* mutant (1021 nspS). The transcriptional expression of *nspS* in strain 1021 was significantly increased in cultures containing either of these polyamines, but not by exogenous cadaverine, 1,3-diaminopropane (DAP), spermidine (Spd) or norspermidine (NSpd). Cell aggregation in liquid cultures did not differ markedly between strain 1021 and 1021 nspS in the presence or absence of PAs. The *S. meliloti* QS-proficient Rm8530 wild-type and *nspS* mutant (Rm8530 nspS) produced similar levels of biofilm under control conditions and 3.2- and 2.2-fold more biofilm, respectively, in cultures with NSpd, but these changes did not correlate with EPS production. Cells of Rm8530 nspS aggregated from two- to several-fold more than the wild-type in cultures without PAs or in those containing Spm. NSpd, Spd and DAP differently affected swimming and swarming motility in strains 1021 and Rm8530 and their respective *nspS* mutants. *nspS* transcription in strain Rm8530 was greatly reduced by exogenous Spm. Bioinformatic analysis revealed similar secondary structures and functional domains in the MbaA proteins of *S. meliloti* and *V. cholerae *, while their NspS proteins differed in some residues implicated in polyamine recognition in the latter species. NspS-MbaA homologs occur in a small subset of soil and aquatic bacterial species that commonly interact with eukaryotes. We speculate that the *S. meliloti* NspS-MbaA system modulates biofilm formation, EPS production and motility in response to environmental or host plant-produced PAs.

## Introduction

The α-proteobacterium *Sinorhizobium meliloti* forms in an economically and agriculturally important nitrogen-fixing symbiosis with plants of the genus *Medicago*. To establish the symbiosis, *S. meliloti* must move through the bulk soil and rhizosphere to the root, synthesize nodulation factors and exopolysaccharides (EPS) and form aggregates or biofilms on the root [1–4]. Later stages of the symbiosis involve penetration by individual *S. meliloti* cells into the root tissue, culminating in the formation of root nodules that house intracellular, nitrogen-fixing *S. meliloti* cells [3]. The symbiotic process as a whole thus involves transitions between motile and biofilm lifestyles [5, 6].

We previously showed that both the ability to synthesize endogenous polyamines (PAs) or the presence of specific PAs in the extracellular environment significantly affect biofilm formation, EPS production and motility in *S. meliloti* Rm8530 [7, 8]. We hypothesized that the phenotypic changes caused by exogenous polyamines were transduced by the *S. meliloti* NspS and MbaA proteins [9, 10]. These proteins are homologs to those constituting the *Vibrio cholerae* NspS-MbaA signalling system, which modulates levels of the second messenger bis-(3′−5′) cyclic diguanosine monophosphate (c-di-GMP) in response to specific environmental PAs [11, 12]. The *V. cholerae* NspS is a periplasmic solute-binding homolog of the PA solute-binding protein PotD and functions as a polyamine sensor. MbaA contains a N-terminal periplasmic domain flanked by two transmembrane regions and tandem GGDEF [diguanylate cyclase (DGC)] and EAL [phosphodiesterase (PDE)] cytoplasmic domains at the C terminus. C-di-GMP is synthesized by DGCs and degraded by phosphodiesterases (PDEs) [13]. In *V. cholerae *, NSpd binding to the periplasmic NspS sensor protein promotes its interaction with the membrane-bound MbaA, increasing the latter’s DGC activity and increasing c-di-GMP levels and thus biofilm formation. Unliganded MbaA present in the absence of NSpd or NspS with bound Spd favours its dissociation from MbaA. This increases MbaA’s PDE activity and lowers c-di-GMP levels and biofilm formation [11, 12].

Bacterial biofilms consist of a self-generated extracellular matrix composed of EPS, proteins and extracellular DNA. Biofilm formation and EPS production by *S. meliloti* and other nodule rhizobia are important not only for symbiosis with legume hosts but also for withstanding abiotic stress conditions encountered in the soil [14–16]. Autoaggregates are a type of biofilm that consist of clumps of bacterial cells that form in liquid cultures and sometimes transform into attached biofilms [17].

EPS production in *S. meliloti* is regulated not only by c-di-GMP but also by quorum sensing (QS). The QS pathway is influenced by c-di-GMP because the production of acylhomoserine lactones (AHLs) is repressed by c-di-GMP. AHL production by the SinI autoinducer synthase is induced by the SinR transcriptional regulator and by the transcriptional regulator ExpR bound to a AHL produced by SinI. ExpR-AHL induces EPS II biosynthesis and that of low MW forms of EPS I. In *S. meliloti *, EPS I, EPS II, arabinose-containing polysaccharide and ß-glucan all participate in biofilm formation and their production is modulated by c-di-GMP and ExpR/SinI. For example, high c-di-GMP levels promote EPS I biosynthesis in *S. meliloti* Rm2011 (*expR* ^-^) but not in Rm8530 (*expR* ^+^). EPS I generally has a negative effect on biofilm formation and cell aggregation, while EPS II promotes their development. The production of EPS II is repressed by high c-di-GMP levels [5, 14, 18, 19].

In this work, we show that both the NspS-MbaA system and QS modulate biofilm formation, EPS production and motility in *S. meliloti* in response to specific extracellular PAs. Bioinformatic analysis revealed that NspS-MbaA systems occur in a restricted number of bacteria that often have pathogenic or mutualistic interactions with eukaryotes. We speculate that *S. meliloti* uses the NspS-MbaA system to modulate motility and EPS and biofilm production in response to polyamines produced by its legume host.

## Methods

### Bacterial strains, plasmids, media and reagents

The bacterial strains and plasmids used in this study are listed in Table 1. Wild-type *S. meliloti* strain Rm8530 is identical to strain 1021 except that it has a functional copy of the transcriptional regulator gene *expR*, which is required for QS [20]. PY (peptone-yeast extract) and LB (Luria broth) complex media and MMSN (minimal medium succinate ammonium) were described previously [7] and solidified with 1.5 % agar when necessary. Bromfield medium containing 0.5 % or 0.3 % Difco Noble Agar (Beckman, Dickinson and Co., Sparks, MD, USA) were prepared as described by Bahlawane [21]. Putrescine ·2HCl [Put; H_2_N(CH_2_)_4_NH_2_], cadaverine [Cad; H_2_N(CH_2_)_5_NH_2_], spermine [Spm; H_2_N(CH_2_)_3_NH(CH_2_)_4_NH(CH_2_)_3_NH_2_], spermidine [Spd; H_2_N(CH_2_)_3_NH(CH_2_)_4_NH_2_], 1,3-diaminopropane [DAP; H_2_N(CH_2_)_3_NH_2_] and norspermidine [NSpd; H_2_N(CH_2_)_3_NH(CH_2_)_3_NH_2_] were purchased from Sigma (St. Louis, MO, USA) and homospermidine·3HCl [HSpd; H_2_N(CH_2_)_4_NH(CH_2_)_4_NH_2_] was obtained from Santa Cruz Biotechnology (Santa Cruz, CA, USA). Aqueous 200 mM PA stock solutions were adjusted to pH 6.8, filter sterilized and added to cultures to a final concentration of 0.1 mM. When required, antibiotics were used at the following concentrations (µg ml^−1^): gentamicin (Gm), 15; kanamycin (Km), 50; spectinomycin (Sp), 100; and streptomycin (Sm), 200.

**Table 1.** Strains and plasmids used in this study

Bacterial strain	Relevant characteristics	Source of reference
*E. coli* DH5α	Strain for cloning	Laboratory collection
* S. meliloti* 1021	Wild-type. Sm^r^ derivative of wild type strain SU47, *expR* ^*-*^ Sm^r^ Nal^r^	[44]
* S. meliloti* Rm8530	Wild-type. *expR* ^+^ derivative of *S. meliloti* 1021, Sm^r^	[20]
* S. meliloti* 1021 nspS	1021 *smc00991*::ΩSp, Sm^r^ Sp^r^	This study
* S. meliloti* Rm8530 nspS	Rm8530 *smc00991*::ΩSp, Sm^r^ Sp^r^	This study
**Plasmids**		
pBBR1MCS-53	∆placZ pBBR1MCS-5 derivative with promoterless *gusA*, Gm^r^	[45]
pBB53nspS::gusA	Transcriptional *smc00991*::*gusA* fusion in pBBMCS-53	This study
pJQ200SK+	Suicide vector for gene replacement, Gm^r^	[46]
pHP45ΩSp	Source of ΩSp element	[47]
pJQ-nspS::ΩSp	*nspS* gene interruped with ΩSp, cloned in pJQ200SK^+^	This study
pRK2013	Helper plasmid, Km^r^	[48]
pTopo	pCR2.1Topo vector for cloning PCR products, Km^r^	Invitrogen
pTopo-nspS	Contains PCR-amplified nspS coding sequence and flanking nt	This study
pTopo-PRnspS	pTopo containing 152 nt of the *nspS* coding sequence and 306 upstream nt	This study

### DNA manipulations

Standard protocols were used to grow *E. coli* and for DNA isolation, restriction digests, cloning and transformation [22]. Bacterial conjugations were done as described previously [7]. DNA sequences were obtained from GenBank (www.ncbi.nlm.nih.gov/gene/). PCR amplifications were done using Dream Taq PCR master mix (Thermo Fisher, Waltham, MA, USA).

### Mutant construction

To inactivate the *S. meliloti* 1021 *nspS* gene, its ORF including 598 and 338 upstream and downstream nt, respectively, was amplified by PCR using primers NspS-F and NspS-R (Table S1, available in the online version of this article). The PCR amplification consisted of an initial denaturing step at 95 °C for 3 min followed by three cycles of 95 °C for 30 s, 56 °C for 30 s, and 72 °C for 2 min, and a final extension at 72 °C for 10 min. The 1.98 kb product was cloned into pTopo to generate plasmid pTopo-nspS (Table 1). The insert from pTopo-nspS was excised with *Sal*I and *Xba*I and ligated into suicide vector pJQ200SK+cut likewise to give plasmid pJQ-nspS. The ΩSp element from pHP45ΩSp was inserted into the unique *Bam*HI site in the *nspS* gene in pJQ-nspS to give pJQ-nspS::ΩSp. This plasmid was conjugated separately into *S. meliloti* 1021 and Rm8530 by triparental mating and double recombinants were selected by sucrose selection [7]. The resulting 1021 *nspS*::ΩSp mutants in the 1021 (*expR* ^-^) and Rm8530 (*expR* ^+^) genetic backgrounds were confirmed by Southern blotting [23] and designated 1021 nspS and Rm8530 nspS, respectively. The insertional inactivation of *nspS* is expected to also prevent the expression of the downstream *mbaA* gene.

### Construction of a *nspS* transcriptional fusion with the β-glucuronidase (*gusA*) gene

The 5′ portion of the *S meliloti nspS* gene and its putative promoter sequence were amplified with primers pNspS-F and pNspS-R (Table S1). The PCR cycling programme included an initial denaturing step at 95 °C for 5 min followed by 35 cycles of 95 °C for 30 s, 54 °C for 30 s, and 72 °C for 1 min, and a final extension at 72 °C for 10 min. The PCR product contained 152 nt of the *nspS* coding sequence and 306 nt upstream of the *nspS* start codon. The product was cloned into pTopo to generate plasmid pTopo-PRnspS. The promoter region-5*′nspS* fragment was excised from pTopo-PRnspS with *Kpn*I and *Xho*I and ligated into vector pBBR1MCS-53 to obtain plasmid pBB53nspS::gusA. The correct transcriptional orientation of the fusion was confirmed by restriction enzyme digestion with *Kpn*I and *Xho*I. The fusion plasmid was transferred to *S. meliloti* 1021 and Rm8530 in separate triparental matings.

### β-glucuronidase (Gus) assays

Cultures of *S. meliloti* 1021 and Rm8530 containing the pBB53nspS::gusA plasmid were grown in MMSN minimal medium without or with 0.1 mM of an exogenous PA for 16 h at 30 °C with shaking at 200 r.p.m. Gus activity was determined by measuring the production of *p*-nitrophenol from the *p*-nitrophenyl β-d-glucuronidase substrate with quantification based on total protein [7]. One unit (U) of activity is defined as the production of 1 nmol of product min^−1^ mg protein^−1^. Strains 1021 and Rm8530 containing pBBR1MCS-53 without an insert lacked Gus activity.

### Motility assays

Single colonies arising on PY plates 3 days after streaking were used as inocula for swarming and swimming motility assays. Swarming was determined by stabbing two or three individual *S. meliloti* colonies from the PY plate into Bromfield medium containing 0.5 % Noble agar without or with 0.1 mM of an exogenous PA using a toothpick and incubating at 30 °C for 72 h. Swarm zones were quantitated by taking the average of two sides of a rectangle that framed the zone [24] using the Macintosh Preview programme rectangular selection tool. Data for these assay is shown in Fig. S1. Swimming motility was assayed by stabbing individual colonies from the PY plate into Bromfield medium containing 0.3 % Noble agar without or with 0.1 mM of an exogenous PA and measuring the diameters of the growth zones on triplicate assay plates after 3 days incubation at 30 °C [8]. Representative results of a swimming assay are shown in Fig. S2.

### Biofilm assays

Biofilm formation by cultures grown in borosilicate glass tubes was determined by crystal violet (CV) staining essentially as described by O´Toole and Kolter [25]. Overnight cultures of *S. meliloti* strains were grown in 50 ml PY medium with appropriate antibiotics and cells were washed twice in MMSN and diluted to an OD_595_ of 0.2. Three millilitres of suspension with or without 0.1 mM of an exogenous PA were added per glass tube and incubated for 72 h at 80 r.p.m., 30 °C. Bacterial growth was quantified by OD_595_ measurement before the removal of the planktonic cells by gentle aspiration. Biofilms were stained with 3 ml 0.1 % CV for 15 min. Tubes were rinsed three times with water and air-dried. The CV was solubilized with 3 ml of 95 % ethanol and the absorbance at 595 nm determined. Biofilm formation was calculated as the A_595_ of the CV solutions divided by the OD_595_ of the cultures.

### EPS quantification

Samples of supernatants of the tube cultures used for biofilm assays were taken to quantify total hexose content by the anthrone method with glucose as a standard [26]. Preliminary experiments showed that quantitation of EPS obtained by precipitation with isopropanol, drying and weighing [27] gave qualitatively similar results to those obtained with the total carbohydrate measurements (results not shown).

### Autoaggregation assays

Autoaggregation assays were done as described by Sorroche *et al*. [28]. Briefly, overnight cultures of *S. meliloti* strains were grown in 50 ml PY medium with appropriate antibiotics and cells were washed twice in MMSN, diluted to an OD_595_ of 0.2 in 50 ml MMSN and grown for 48 h. Five ml of bacterial suspension was transferred to a glass tube (12×100 mm) and allowed to settle for 24 h at 4 °C. A 0.2 ml aliquot of the upper portion of suspension was carefully transferred to a 96-well microplate and the OD_600_ was measured (OD_final_). A control tube was vortexed for 30 s, and the OD_600_ was determined (OD_initial_). The percentage of aggregation was calculated as 100[1-(OD_final_/OD_inital_].

### Bioinformatics

Phylogenetic analysis was done by separate blastP searches of the *S. meliloti* and *V. cholerae* NspS-MbaA protein region against 16 076 complete proteobacterial genomes from GenBank that had identical GCA (GenBank assemblies) and GCF (RefSeq assemblies) annotations. Hits to NspS-MbaA homologs with at least 60 % sequence coverage and 30 % amino acid identity to the query sequences and in which *nspS* and *mbaA* were separated by ≤20 bp were included in the phylogeny. The phylogenetic analysis was done with PhyML 3.3.20190321 [29]. Multiple sequence alignments were made with Clustal Omega at the EMBL’s European Bioinformatics Institute website (https://www.ebi.ac.uk/Tools/msa/clustalo/). Operon prediction for the *S. meliloti* 1021 *nspS-mbaA* was made using the Operon and Regulon feature with *smc00991* as query at http://www.microbesonline.org. The presence of putative transmembrane segments in protein sequences was determined with DeepTMHMM ([30]; https://dtu.biolib.com/DeepTMHMM).

### Statistical analysis

Experiments were repeated at least three times with three to four technical replicates for each treatment. One-way analysis of variance and Tukey post-hoc test were used to identify statistically significant differences (*P*<0.05) using GraphPad Prism, version 5.01.

## Results

### Sequence and phylogenetic analysis of the *S. meliloti nspS-mbaA*

The *S. meliloti* 1021 *nspS* and *mbaA* genes (*smc00991* and *smc00992*, respectively) overlap by 3 bp and the two genes are predicted to form an operon (see Methods). The protein products of these *S. meliloti* genes are henceforth referred to as Sm NspS and Sm MbaA. In *V. cholerae* MO10, *nspS* (*vc0704*) overlaps the downstream *mbaA* gene (*vc07003*) by 16 bp and the genes are cotranscribed [31]. The *V. cholerae* NspS and MbaA proteins are henceforth designated Vc NspS and Vc MbaA.

**Fig. 1.**
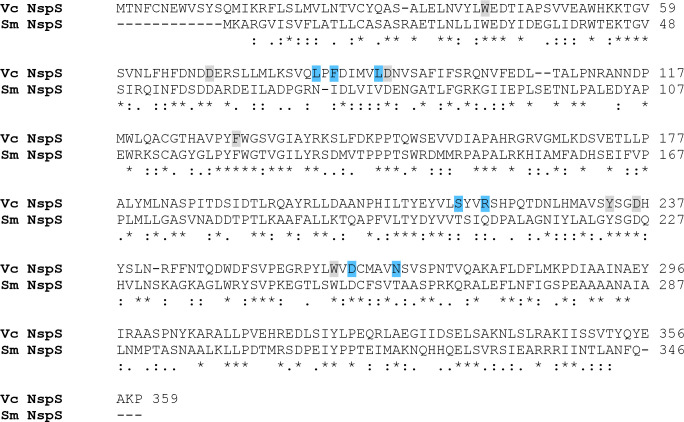
Clustal Omega alignment of NspS proteins from *V. cholerae* O1 (Vc NspS) and *S. meliloti* 1021 (Sm NspS). Identical residues, conserved substitutions and semi-conserved substitutions are indicated by asterisks, colons and periods, respectively. Residues outlined in grey in the Vc NspS are those predicted to make direct contact with NSpd and blue-shaded residues cause biofilm phenotypes that are non-responsive to NSpd if mutated [32]. The percentage of identity/similarity was 31.95/52.06 calculated by the BLOSSUM62 matrix. The alignment was done with ClustalW.

The Sm NspS and Vc NspS have nearly 32 % amino acid identity and 52 % similarity (Fig. 1) and both proteins have significant similarity to bacterial spermidine/putrescine ABC transporter substrate-binding proteins PotD and PotF. Homology modelling of the Vc NspS using the *Pseudomonas aeruginosa* PotD as a template predicted that eight residues directly contact NSpd in the ligand-binding pocket (W41, D70, D90, F131, Y233, D236,W261 D263) [32]. These residues are all conserved in the Sm NspS while only five of the eight (W41, Y233, D236, W261, D263) are identical or conservatively substituted in six other representative PotD homologs analysed by Young *et al*. [32], including PotD and PotF from *E. coli*. Of seven amino acids shown by random mutagenesis to be required for the Vc NspS-mediated biofilm response to NSpd, only those corresponding to L89, S216, D263 are conserved or similar in the Sm NspS [32] (Fig. 1).

**Fig. 2.**
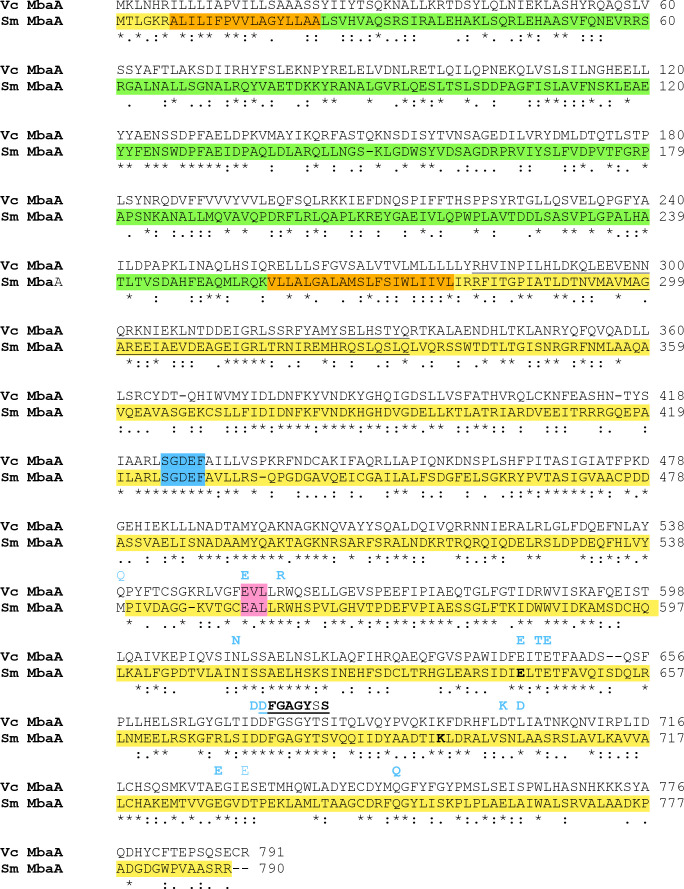
Clustal Omega sequence alignment of the *V. cholerae* O1 (Vc MbaA) and *S. meliloti* 1021 (Sm MbaA) MbaA proteins. Identical residues, conserved substitutions and semi-conserved substitutions are indicated by asterisks, colons, or periods. Colour shading of residues in the Sm MbaA denotes protein topology predicted with DeepTMHMM [30]: yellow, cell interior; orange, membrane, green, outside of membrane (periplasm). The cyclase and phosphodiesterase motifs are indicated in blue and pink, respectively. A putative HAMP motif present in both proteins first reported by Schäper *et al*. [35] was localized using Prosite and is underlined. The 14 key amino acid residues required for PDE activity in the *P. aeruginosa* RocR protein [36] appear in light blue above the sequences: the 12 residues in bold are present in Sm MbaA. The domain consisting of eight amino acids is shown above the sequences and underlined is the functionally important loop six identified in RocR [49], and residues identical to those in the Sm MbaA are in bold.

The Sm MbaA and Vc MbaA amino acid sequences are 30.9 % identical (50.1 % similar) and both proteins have an alternative DGC motif (SGDEF) in place of the canonical GG[D/E]EF (Fig. 2). DGC motifs in which a different amino acid replaces the first G are relatively common and are functional in c-di-GMP synthesis [33, 34]. Both the Vc and Sm MbaAs lack a nearby allosteric inhibition site (RXXD), which is also absent in the majority of the putative DGCs in *S. meliloti* [35]. A putative HAMP domain, found in many signal tranducing regulatory proteins, occurs upstream of the SGDEF motif in both MbaA proteins (underlined in Fig. 2). The Sm MbaA has a canonical PDE motif (EAL) (Fig. 2) while the Vc MbaA has EVL. The four residues involved in binding Mg^2+^ in the *P. aeruginosa* RocR PDE (D295, N233, E265 and E175 [36]) are conserved in the *S. meliloti* and *V. cholerae* MbaAs (as D674, N611, E643 and E552 in the Vc MbaA). A number of other RocR catalytic residues that are conserved in Sm MbaA are described in Fig. 2

The predicted secondary structure of the Sm MbaA consists of N-terminal amino acids 1–6 localized in the cytoplasm, a transmembrane domain spanning residues 7–23, periplasmic residues 24–256, a second transmembrane segment from residues 257–277 and cytoplasmic residues 278–787. The cytoplasmic segment of MbaA (amino acids 278–787) contains the HAMP, cyclase and PDE domains (Fig. 2). The Vc MbaA predicted secondary structure is very similar [32].

blastP analysis of 16 076 complete proteobacterial genomes using the *S. meliloti* 1021 NspS-MbaA proteins as query returned 222 genomes that encoded the proteins. The same search using the *V. cholerae* NspS-MbaA as query returned just eight fewer (216 total) genomes, which demonstrates very similar results with either query. Thus, among proteobacteria, NspS-MbaA paralogs occur in less than 1.4 % of the genomes analysed.

**Fig. 3.**
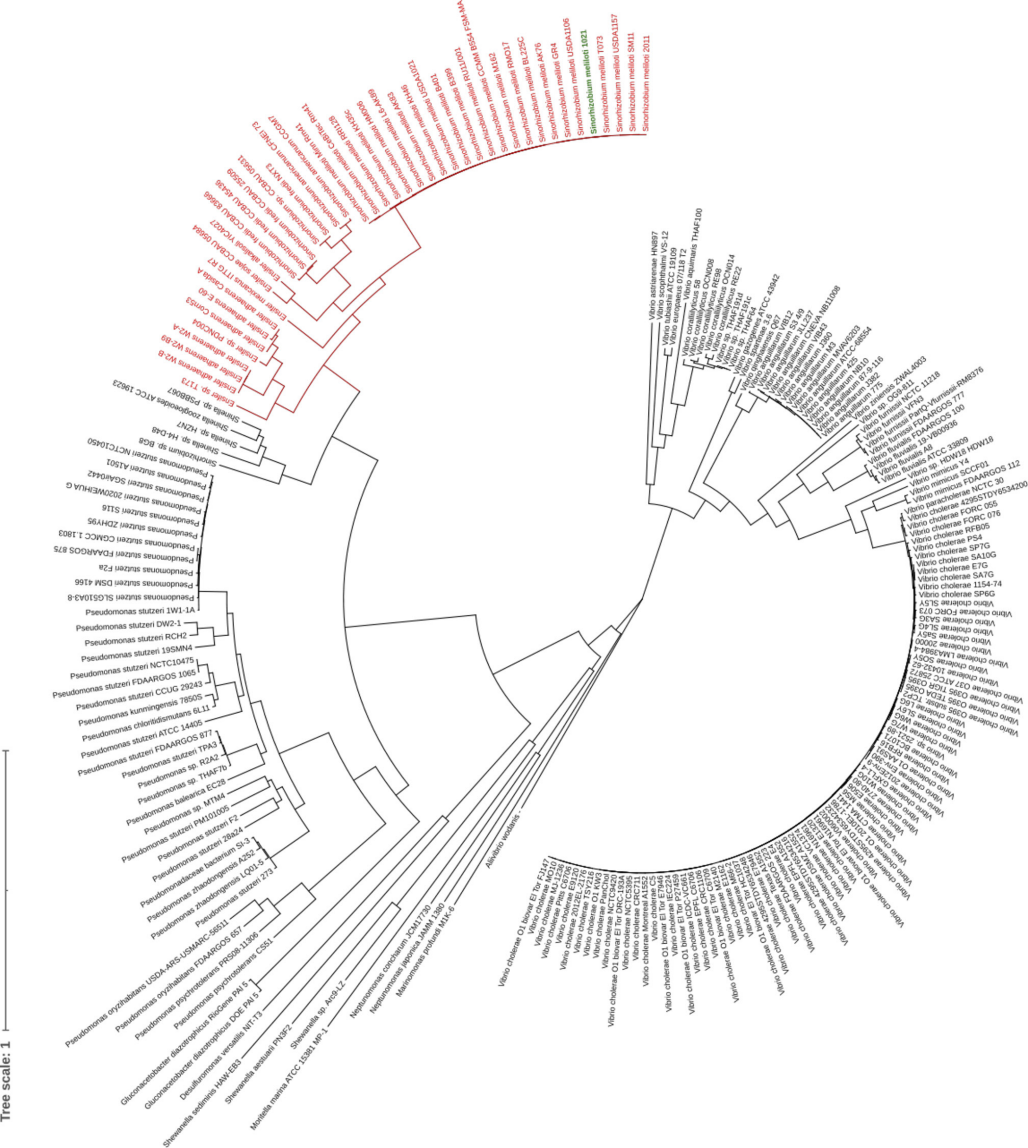
Phylogenetic analysis based on 222 proteobacterial genomes that contain neighbouring genes encoding NspS-MbaA. Hits were returned by blastP search using the *S. meliloti* NspS-MbaA proteins as query against 16 076 complete proteobacterial genome sequences from NCBI. *Sinorhizobium* and *Ensifer* species are in red text, *S. meliloti* 1021 is in green.

Phylogenetic analysis of the potential NspS-MbaA operons resolved major clades into which *Vibrio *, *Pseudomonas* and *Sinorhizobium */* Ensifer* species were grouped (Fig. 3). Smaller clades were formed by *Marinomonas *, *Shewanella *, and *Shinella* spp., among others, perhaps because there are fewer complete genome sequences available for these genera. Many of the organisms present in the phylogeny are either aquatic and associated with animal hosts (* Vibrio* spp.) or are found in soil and associated with plants (* Sinorhizobium */* Ensifer *). Among rhizobia, NspS-MbaA occurs in 24 *S *. *meliloti* strains, all isolated from *Medicago* spp. Four *Sinorhizobium fredii* strains isolated from *Phaseolus* or *Glycine* contain the proteins. Most *Ensifer* species having NspS-MbaA were isolated from legume nodules, while *Ensifer adherens* strains were mostly from soil. There is a 3 bp overlap of *nspS* and *mbaA* in these rhizobia.

Over 180 strains of environmental (mostly water) and clinical isolates of *V. cholerae* contain NspS-MbaA. Other *Vibrio* species in the phylogeny were mostly isolated from fresh- or salt-water animals. *nspS* and *mbaA* overlap by 16 bp in all *Vibrio* species.

Most of the 24 of *Pseudomonas stutzeri* strains form a cluster that contains isolates from sediments, sludge, soil and rhizospheres, and seawater. Other *Pseudomonas* species represented by both environmental and clinical isolates form distinct clades nearby. Plant-associated *Pseudomonas* species like *P. fluorescens *, *P. syringae* and *P. putida* are absent from the phylogeny. A plant growth-promoting rhizobacterium isolated from sugarcane roots, *Gluconacetobacter diazotrophicus* PA1 5, is grouped with the Pseudomonas clades. The *nspS* and *mbaA* genes overlap by 13 bp in *Pseudomonas* species and *G. diazotrophicus,* and in a few *Shinella *, *Neptunomonas *, *Marinomonas* and *Allivibrio* isolates that flank the *Pseudomonas* clades. Of the species included in the phylogeny, the *nspS* and *mbaA* genes do not overlap and are separated by 3 to 13 nt in *Desulfuromonas versatilis, Moritella marina* (one strain each) and *Shewanella* species (three strains).

The synteny of genes encoded near *nspS-mbaA* was perfectly conserved in 41 of the 42 S. *meliloti* strains and conserved to a large degree in other *Sinorhizobium* and *Ensifer* species (Fig. S3). Genes encoded near the *S. meliloti nspS-mbaA* did not bear any apparent relation to PA or c-di-GMP metabolism. A similar analysis done with the *V. cholerae* strains showed that all had a largely syntenic set of genes with no obvious relation to NspS-MbaA function, and these genes were unrelated to those neighbouring *nspS-mbaA* in *S. meliloti *.

In summary, the proteobacteria that encode *nspS* and *mbaA* include a restricted subset of aquatic or soil dwelling species that often have pathogenic or mutualistic interactions with eukaryotes.

### Growth of the *S. meliloti* wild-types and *nspS* mutants

For the phenotypic characterization experiments described below, cultures of the 1021 and Rm8530 wild-types and *nspS* mutants were grown in MMSN without or with an exogenous PA (0.1 mM). There were no large differences in the growth of the strains with or without PAs (Figs S4 and S5).

### Effect of exogenous PAs on biofilm formation, EPS production and autoaggregation in QS-deficient strain 1021

**Fig. 4.**
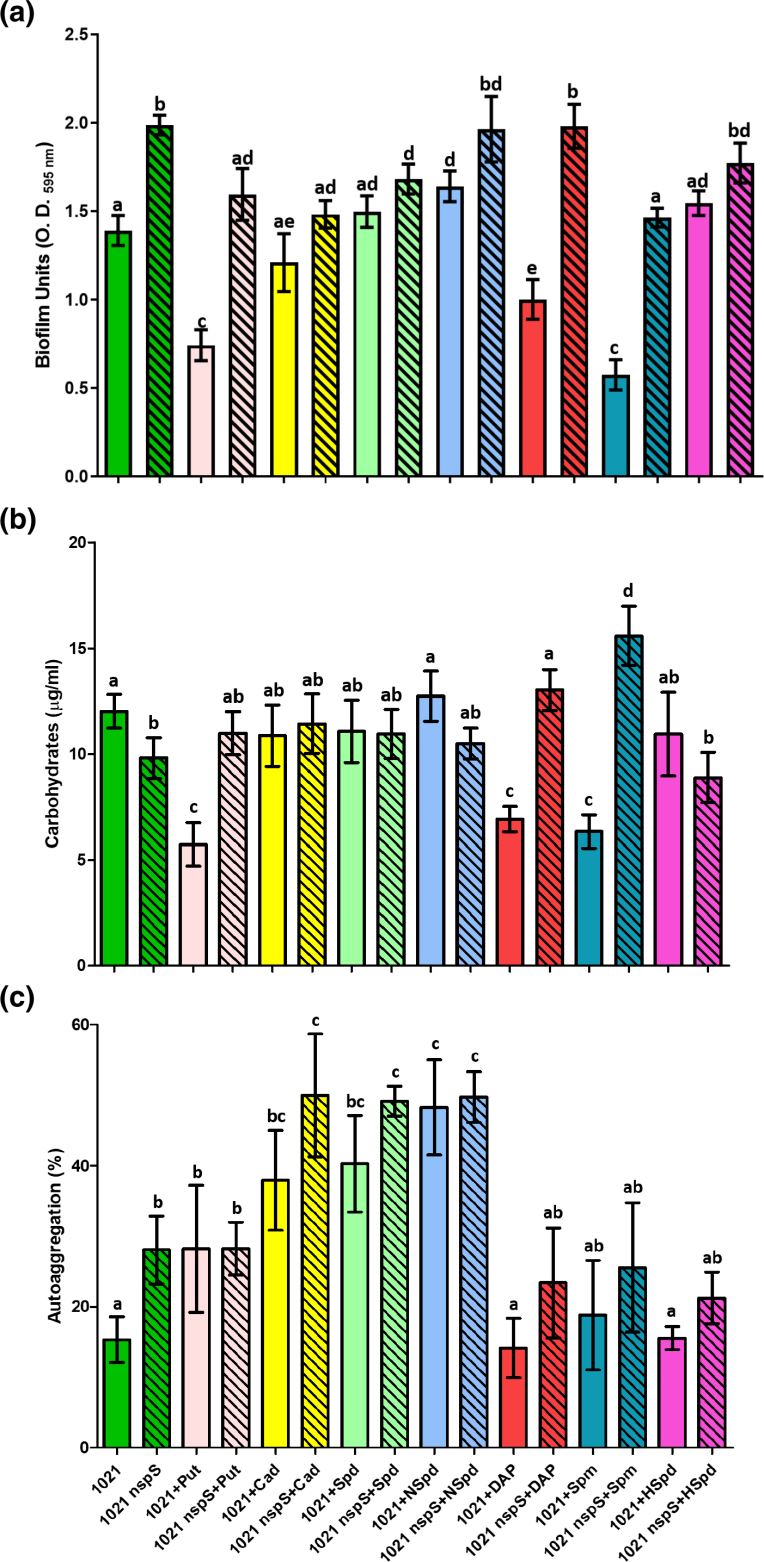
Biofilm formation (a), culture supernatant carbohydrates (b) and autoaggregation (c) of *S. meliloti* 1021 and 1021 nspS grown in the absence or presence of exogenous (0.1 mM) PAs. Biofilm was determined by CV staining of cultures grown in borosilicate glass tubes. Carbohydrate (hexose) concentrations of culture supernatants from biofilm assay cultures were determined by the anthrone method and represent total EPS production. Autoaggregation assay show the mean percentage aggregation for each strain. The experiments were performed in quadruplicate and repeated three times. Error bars represent sd. One-way analysis of variance with a Tukey post-test were performed. Values for columns with the same letter are not statistically significantly different from each other at *P*<0.05. The OD_initial_ absorbances for the control culture replicates from all of the experimental treatments was 1.53±0.13.

To determine the effect of NspS on biofilm formation in *S. meliloti* 1021, which lacks a functional QS system, we grew the wild-type and 1021 nspS in MMSN without (control conditions) or with an exogenous PA and determined biofilm formation after 3 days (Fig. 4a). In comparison to wild-type 1021 grown under control conditions, cultures with exogenous Spm, Put, and DAP had 59, 47 and 28 % less biofilm, respectively. These reductions in biofilm formation were statistically significant (*P*<0.05). More biofilm was formed by 1021 nspS in comparison to the wild-type under all conditions, with the differences being significant (*P*<0.05) for the control and Put, DAP and Spm-containing cultures. Under control conditions, 1021 nspS made 1.4-fold more biofilm than the wild-type (Fig. 4a), which is significant at *P*<0.05. In comparison to the control, exogenous NSpd increased biofilm formation by the 1021 wild-type by a statistically significant 1.2-fold (*P*<0.05). In 1021 nspS, added NSpd, DAP or HSpd did not change the high level of biofilm formation seen under control conditions. Biofilm formation in 1021 nspS cultures containing Put, Cad, Spd or Spm decreased a statistically significant 15–25 % (*P*<0.05) relative to the control culture. In summary, when *nspS* is inactivated in strain 1021, biofilm levels show less change in response to exogenous PAs.

To relate total EPS production with biofilm formation, we measured the hexose content of culture supernatants obtained from the cultures used for the biofilm assays. EPS levels in wild-type 1021 were only statistically significantly affected in cultures containing exogenous Put, Spm and DAP, which reduced EPS levels a statistically significant (*P*<0.05) 53, 47 and 43 % relative to the control culture (Fig. 4b). Compared to control conditions, EPS production by 1021 nspS increased 1.3- and 1.6-fold in cultures containing DAP and Spm, respectively. EPS production by the wild-type in comparison to the *nspS* mutant increased in the mutant grown with Put, DAP or Spm (1.9- to 2.4-fold), and decreased 19 % under control conditions. All of these changes were statistically significant at *P*<0.05. In summary, the levels of biofilm formation and EPS production correlated with the significant changes seen in these parameters for both 1021 wild-type and *nspS* mutant in cultures containing Put, DAP and Spm (Fig. 4a, b).

We measured autoaggregation in 1021 wild-type and 1021 nspS cultures grown in the absence or presence of an exogenous PA (Fig. 4c). Under control conditions without added PAs, 1021 nspS aggregated 1.8-fold more than the wild-type (significant at *P*<0.05; Fig. 4c), consistent with a 1.4-fold increase in biofilm formation (Fig. 4a) but contrasted by a 19 % decrease in EPS production (Fig. 4b). In cultures with added Put versus control conditions, aggregation increased 1.8-fold (significant at *P*<0.05) in both the wild-type and 1021 nspS (Fig. 4c), which did not reflect the significantly increased biofilm and EPS production in the mutant over wild-type when grown with Put (Fig. 4a, b). Although the 1.7- and 1.4-fold increases in aggregation in the mutant versus wild-type grown with DAP and Spm, respectively (Fig. 4c) are not statistically significant (*P*<0.05), they do correspond to the mutant’s increased biofilm and EPS production under these conditions (Fig. 4a, b).

In comparison to control conditions, Cad, Spd and NSpd each increased autoaggregation by 1.7- to 1.8-fold in the mutant and by 2.4- to 3.1-fold in the wild-type (significant at *P*<0.05; Fig. 4c).

### Effect of exogenous PAs on biofilm formation, EPS production and aggregation in QS-competent strain Rm8530

**Fig. 5.**
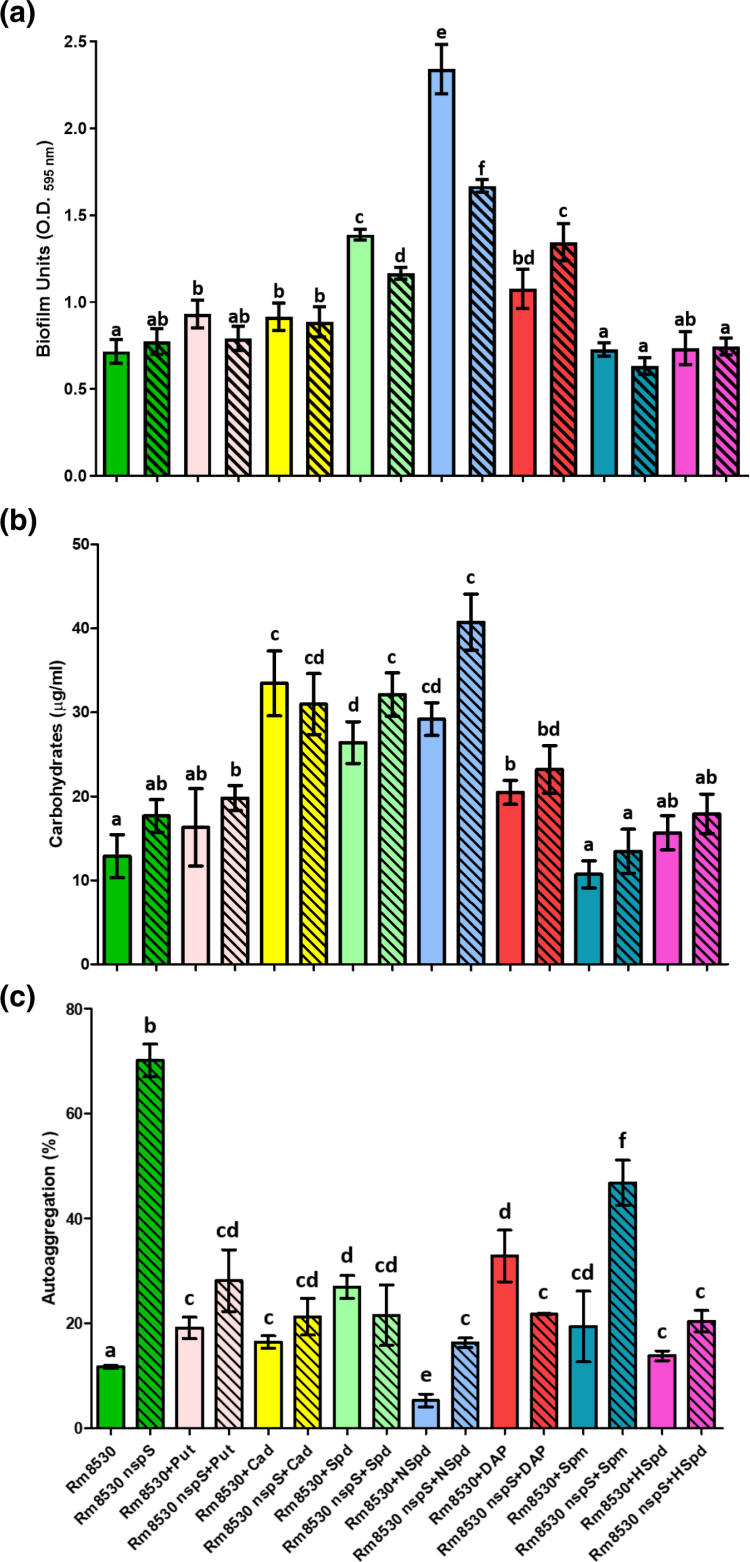
Biofilm formation (a), culture supernatant carbohydrates (b) and autoaggregation (c) of *S. meliloti* Rm8530 and Rm8530 nspS grown in the absence or presence of exogenous (0.1 mM) PAs. Biofilm was determined by CV staining of cultures grown in borosilicate glass tubes. Carbohydrate (hexose) concentrations of culture supernatants from biofilm assay cultures were determined by the anthrone method and represent total EPS production. Autoaggregation assay show the mean percentage aggregation for each strain. The experiments was performed in quadruplicate and repeated three times. Error bars represent sd. One-way analysis of variance with a Tukey post-test were performed. Values for columns with the same letter are not statistically significantly different from each other at *P*<0.05. The OD_initial_ absorbances for the control culture replicates from all of the experimental treatments was 1.01±0.13.

To determine how QS affected the biofilm, EPS and aggregation responses of *S. meliloti* to PAs and the NspS-MbaA system, we repeated the phenotypic assays described above with strains Rm8530 and Rm8530 nspS (Fig. 5). Under control conditions and in cultures with exogenous Put, Cad, Spm or HSpd, biofilm formed at a similar level and there were no large differences between strains Rm8530 and Rm8530 nspS. In cultures with added Spd, biofilm formation was 1.9- and 1.5-fold higher than the control level in the wild-type and mutant, respectively, while NSpd caused the biggest changes in biofilm formation with 3.3- and 2.2-fold increases in Rm8530 and Rm8530 nspS, respectively, versus controls. Added DAP caused 1.5- and 1.8-fold increases in the Rm8530 and Rm8530 nspS, respectively (Fig. 5a). All of these changes were significant at *P*<0.05.

**Fig. 6.**
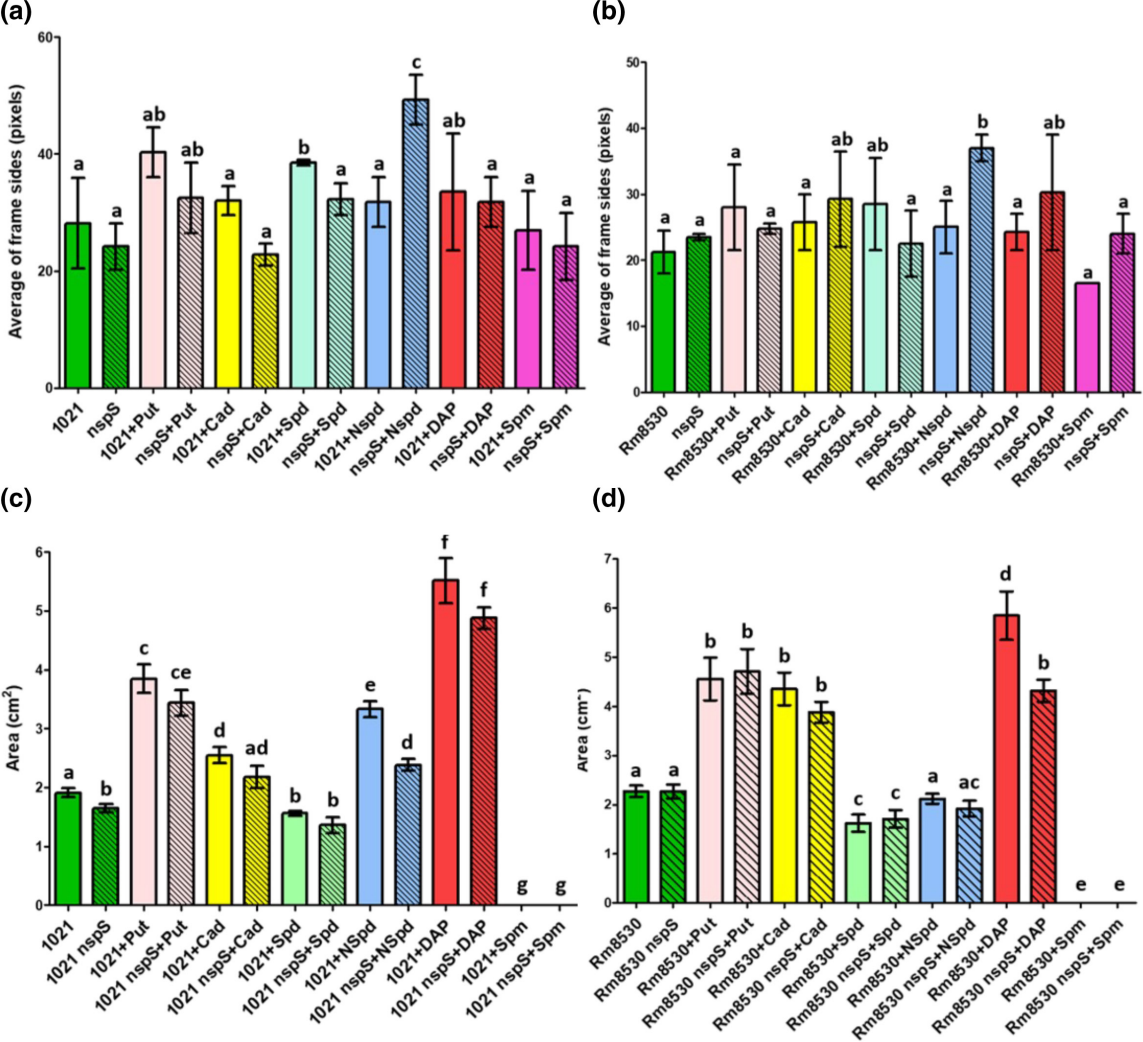
Swarming motility of *S. meliloti* 1021 and 1021 nspS (a), Rm8530 and Rm8530 nspS (b) grown without or with exogenous (0.1 mM) PAs. The experiment was performed in triplicate and repeated three times. Swimming motility of *S. meliloti* 1021 and 1021 nspS (c), Rm8530 and Rm8530 nspS (d) grown without or with exogenous (0.1 mM) PAs. The experiment was performed in triplicate and repeated three times. Error bars represent sd. One-way analysis of variance with a Tukey post-test were performed. Values for columns with the same letter are not statistically significantly different from each other at *P*<0.05.

Hexose assays showed that under control conditions Rm8530 nspS produced 1.4-fold more EPS than the wild-type (Fig. 5b). Although not statistically significant (*P*<0.05), this increase in EPS production corresponded to a 1.1-fold increase in biofilm (Fig. 6a). Relative to control conditions, EPS production in the wild-type and mutant changed 2.3- and 2.3-fold with NSpd, 2.7- and 1.8-fold with Cad, 2.1- and 1.8-fold with Spd, 1.6- and 1.3-fold with DAP, respectively. (Fig. 5b). These changes were significant (*P*<0.05) except for the 1.3-fold value. Cultures with exogenous Spd or NSpd showed an inverse correlation between biofilm and EPS (Fig. 5a, b).

Cell aggregation increased 2.4- to 5.8-fold in Rm8530 nspS versus the wild-type in control cultures or with exogenous NSpd or Spm (Fig. 5c). These large differences (*P*<0.05) between wild-type and mutant were not reflected in similar changes in biofilm formation. Exogenous NSpd greatly decreased (*P*<0.05) autoaggregation in both strains while substantially increasing EPS production and biofilm formation. DAP caused a significant (*P*<0.05) 31 % decrease in autoaggregation in the mutant versus the wild-type. Changes in autoaggregation between Rm8530 and Rm8530 nspS in cultures grown with Put, Cad or Spd were statistically non-significant (Fig. 5c).

### The NspS-MbaA system alters motility in response to exogenous PAs

Swarming is a social spreading phenomenon involving cell elongation and hyperflagellation that occurs on solid or semisolid surfaces such as the culture medium containing 0.5 % agar used in our assays. Swarming by wild-type strain 1021 increased 1.4-fold with Spd relative to control conditions (*P*<0.05; Fig. 6a). Swarming by 1021 nspS increased twofold with added NSpd in comparison to control conditions. (*P*<0.05; Fig. 6a).

In comparison to control conditions, swarming in Rm8530 was not significantly affected by PAs (Fig. 6b). For Rm8530 nspS, NSpd increased swarming 1.6-fold. Swarming in the mutant versus wild-type was increased 1.5-fold by exogenous NSpd (both increases statistically significant at *P*<0.05; Fig. 6b).

Swimming motility involves the movement of individual cells in liquid or semisolid media such as one with 0.3 % agar, as used in our assays. In 1021 and 1021 nspS, exogenous PAs with the exception of Spd increased swimming 1.4- to 3.1-fold (all significant at *P*<0.05). Spd decreased swimming by about 20 % in both strains, but this was only significant (*P*<0.05) for the wild-type (Fig. 6c). The main difference between the two strains occurred with exogenous NSpd, which decreased swimming in the mutant significantly (nearly 30 %, *P*<0.05) in comparison to the wild-type. Exogenous Spm completely prevented swimming in both the wild-type and mutant strains (Fig. 6c).

Swimming in Rm8530 and Rm8530 nspS was identical under control conditions and increased significantly (*P*<0.05) 1.9- to 2.6-fold in both strains grown with Put, Cad or DAP (Fig. 6d). Swimming was reduced significantly (25 %, *P*<0.05) in the mutant as compared to the wild-type in cultures with added DAP. Swimming was completely prevented in both strains by added Spm (Fig. 6d).

In summary, the wild-type strains and their respective nspS mutants usually responded similarly to added PAs. However, the results of swimming versus swarming assays differed with respect to how PA supplementation affected motility in comparison to the control conditions.

### Exogenous polyamines affect *nspS* transcription differently in 1021 and Rm8530

**Fig. 7.**
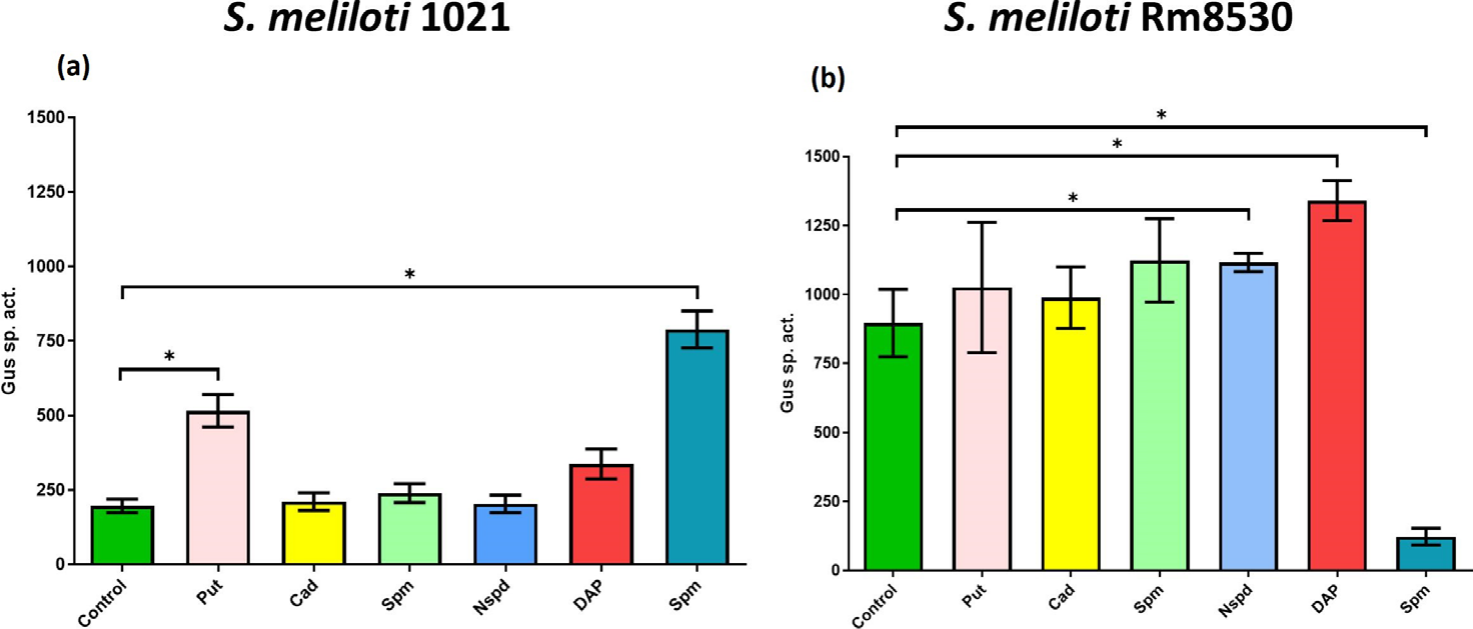
β-glucuronidase (Gus) activities produced by *S. meliloti* 1021/pBBR53nspS::gusA (a) and Rm8530/pBBR53nspS::gusA (b) grown with exogenous PAs. Cultures were grown 18 h at 30 °C in MMSN minimal medium with the indicated PA at a 0.1 mM. Values are the mean±sd for two independent experiments with two technical replicates. 1 U=nmol product min^−1^ mg protein ^−1^. One-way analysis of variance with a Tukey post-test were performed. Significance bars and asterisks indicate treatments differing from the control with *P*<0.05.

The effect of exogenous PAs on transcription of *nspS* was measured in strains 1021 and Rm8530 with a plasmid containing the *nspS* promoter region fused to the *gusA* reporter gene. Growth of the 1021 fusion strain with exogenous Put and Spm caused 2.5- and 3.9-fold increases in *nspS* transcription, respectively. No changes in *nspS* expression occurred when Cad, Spd or NSpd were added to the cultures (Fig. 7a). In Rm8530, small but statistically significant changes (*P*<0.05) in *nspS* expression occurred with exogenous NSpd and DAP (1.25- and 1.5-fold increases, respectively), and Spm, which lowered *nspS* expression to 14 % of the control level (Fig. 7b).

## Discussion

We previously reported that exogenous PAs affect biofilm formation, EPS production and motility in *S. meliloti* Rm8530 [7, 8]. We hypothesized that these effects were at least partly due to the *S. meliloti* NspS-MbaA homologs acting as a PA sensing-transducing system in a manner analogous to the *V. cholerae* NspS-MbaA proteins [9, 10]. We show here that the inactivation of the *S. meliloti* NspS-MbaA system does indeed affect the ability of specific exogenous PAs to alter biofilm formation, EPS production and motility in *S. meliloti* 1021 and Rm8530 and that the phenotypic responses are also conditioned by QS.

The role of the *S. meliloti nspS* has not been previously studied, but *mbaA* null mutants have been characterized. Schäper *et al*. [36] found that a *S. meliloti* Rm2011 *mbaA* mutant made 1.17-fold more biofilm and had 0.84-fold the swimming motility as the wild-type. Rm2011 is an *expR* ^-^ strain closely related to 1021. Wang *et al*. [37] found that a *S. meliloti* 1021 *mbaA* mutant swarmed significantly less and produced several fold more EPS than the wild-type. Biofilm formation was not reported [37]. In strain 1021, we found a similar trend (more biofilm and/or EPS and lower motility) when *nspS* was inactivated (Fig. 4a, b). As mentioned (see Methods), the insertional inactivation of *nspS* almost certainly prevents *mbaA* expression*,* effectively making our *S. meliloti nspS* mutants *nspS mbaA* double mutants.

The overlap of *nspS* and *mbaA* might allow translational coupling of the two genes [38]. In *V. cholerae *, *nspS* overlaps *mbaA* by 16 bp and the genes are co-transcribed [31]. The great majority of the *nspS* and *mbaA* gene pairs obtained in our phylogenetic analysis (Fig. 3) overlap and are perhaps co-regulated, thus allowing balanced production of the two proteins forming the system.

In the QS-deficient strain 1021 wild-type, exogenous Put, DAP and Spm all greatly reduce biofilm formation, while 1021 nspS showed little response to these PAs and maintained a high level of biofilm formation in their presence (Fig. 4a). Changes in biofilm formation in response to different exogenous PAs in 1021 and 1021 nspS correlated with total EPS production. It is notable that Put, DAP and Spm were the only PAs tested that affected the transcriptional expression of *nspS* in strain 1021, causing 1.7- to 3.9-fold increases (Fig. 7). Based on the *V. cholerae* model, we hypothesize that the *S. meliloti* NspS that is unligated to a PA (apo-NspS) or ligated to Put, DAP or Spm does not interact with MbaA and permits MbaA to have full PDE activity, thus lowering c-di-GMP levels, EPS production and biofilm formation. Having more copies of NspS-MbaA due to the increased transcription of *nspS* (and presumably the overlapping *mbaA*) would result in higher total PDE activity and c-di-GMP degradation in the presence of these PAs.

Cell autoaggregation was markedly affected by exogenous PAs but did not greatly differ between the 1021 wild-type and *nspS* mutant (Fig. 4c). Aggregation did not correlate with biofilm formation (Fig. 4a), contrary to previously reported interdependence of these phenotypes in *S. meliloti* 1021 [4, 28]. The discrepancy could be due to methodological differences between these experiments.

In the QS-proficient Rm8530 genetic background, exogenous PAs affected biofilm formation to a lesser degree than in strain 1021. Exogenous NSpd and DAP increased biofilm formation in the Rm8530 wild-type and *nspS* mutant. In cultures with NSpd, the wild-type formed more biofilm than the mutant, while cultures with DAP had the opposite pattern. (Fig. 5a). These results indicate a partial dependence on NspS for changes in biofilm formation. NSpd and DAP were also the only PAs to significantly increase *nspS* transcription in Rm8530. Increased biofilm formation in response to NSpd resembles the biofilm-enhancing effect of this PA in *V. cholerae* [29]. NSpd production is relatively rare in bacteria, occurring mostly in *Vibrio* species and, among rhizobia, in *Sinorhizobium* species [10, 39, 40]. As in *V. cholerae *, its presence in the environment could act as a signal of ‘self’ and thus promote biofilm formation by *Sinorhizobium* spp.

The *S. meliloti nspS* mutants are expected to also lack *mbaA* expression (see above). In *V. cholerae *, *nspS mbaA* double mutants make more biofilm than the wild-type [12], similar to the higher biofilm formation in both the Rm8530 wild-type and *nspS* mutant grown with NSpd. Total EPS production did not correlate with biofilm levels in the Rm8530 wild-type and mutant strains (Fig. 5b). While this contrasts the EPS-biofilm correlation observed in strain 1021, it is not wholly unexpected given the complex effects that different mixtures of EPS I, EPS II, arabinose-containing polysaccharide and ß-glucan have on biofilm formation [18]. As described in the Introduction, the production of all these polymers is affected by changes in c-di-GMP levels. In comparison to the wild-type, Rm8530 nspS showed very high autoaggregation in cultures without exogenous PAs (Fig. 5c). NSpd, which increased biofilm formation in both the wild-type and mutant, greatly decreased autoaggregation in both strains. As measured in our assays, biofilm formation and autoaggregation were not correlated in either 1021 or Rm8530 wild-types or *nspS* mutants, with each wild-type and mutant showing a distinct patterns. The contrasting results regarding exogenous PAs affecting c-di-GMP-dependent phenotypes in strains 1021 and Rm8530 could reflect QS being placed higher in the regulatory hierarchy and overriding NspS-MbaA [41].

Within the 1021 and Rm8530 genetic backgrounds, swarming motility was significantly affected in the wild-types versus *nspS* mutants only in assays with NSpd, where the inactivation of *nspS* causes an increase in swarming motility relative to the wild-type (Fig. 6a and b). Changes in swarming motility did not correlate with biofilm, EPS or autoaggregation in any of the strains. Swimming motility showed no large *nspS*-dependent differences in either 1021 or Rm8530, although significant changes in swimming were caused by some PAs (e.g. DAP caused notable increases in both of the wild-types and *nspS* mutants) (Fig. 6c, d). In summary, changes in swimming and swarming motility caused by exogenous PAs were generally similar in the 1021 and Rm8530 wild-types and their corresponding *nspS* mutants. This is consistent with previous results indicating that c-di-GMP has less effect on motility than biofilm in *S. meliloti* [18].

We did not find any PAs that reciprocally regulated biofilm formation or other phenotypes in *S. meliloti *. This contrasts *V. cholerae *, where NSpd and Spd or Spm significantly increase and decrease, respectively, the formation of biofilm and expression of EPS biosynthesis genes [11, 12, 42]. The modulation of c-di-GMP levels and biofilm formation by the NspS-MbaA system in *V. cholerae* is exerted through the absolute and proportional extracellular concentrations of the signal PAs NSpd and Spd [12]. It is possible that changes in biofilm formation dependent on the NspS-MbaA system in *S. meliloti* would differ with higher or lower concentrations of PAs than those used in our experiments or with mixtures of different PAs.

We found that many of the amino acid residues required for the function of PotD/PotF homologs and PDE and/or DGC catalysing proteins were conserved in the corresponding *S. meliloti* NspS or MbaA proteins. The *S. meliloti* proteins also conserved many residues specific to the function of the *V. cholerae* NspS and MbaA (Figs 1 and 2). These sequence similarities between the two systems support that the *S. meliloti* NspS-MbaA is functional sensor-transducer system, while the differences in some key residues could explain the different phenotypic responses of the *S. meliloti* and *V. cholerae* systems. NspS proteins have their greatest sequence similarity to periplasmic binding proteins of the ABC transporters for Spd and Put, PotD and PotF, respectively [43].

The taxonomic distribution of NspS-MbaA proteins in proteobacterial genomes indicates that it is present in relatively few species, which mostly inhabit water or soil. Most of these species interact symbiotically or pathogenically with eukaryotes, and their NspS-MbaA systems might respond to environmental PAs released by these organisms in order to modulate the interaction.

In conclusion, the *S. meliloti* NspS-MbaA affects several c-di-GMP-dependent phenotypes in response to exogenous PAs. We are currently determining how global gene expression is affected by PAs in the *S. meliloti* wild-types and *nspS* mutants. We will also determine if the *S. meliloti* NspS-MbaA system responds to PA cues and tailors biofilm formation and other c-di-GMP phenotypes to specific environments, including symbiosis.

## Supplementary Data

Supplementary material 1
