# Supplementary material for: The Sinorhizobium meliloti NspS-MbaA system affects biofilm formation, exopolysaccharide production and motility in response to specific polyamines
Source: Microbiology (Reading). 2023 Jan 30;169(1):001293. doi: 10.1099/mic.0.001293 (PMC9993111; doi:10.1099/mic.0.001293)
Supplement: Supplementary material 1 [file mic-169-1293-s001.pdf]

Table S1. Oligonucleotide primers used in this work.

Primer name	5'-3' nucleotide sequence	Description
NspS-F	CCTTCTTCGTCgaCGAAAGG	Forward primer for *nspS* cloning for mutagenesis. Introduced nt (lower case) produce a *Sal*I site (underlined).
NspS-R	GTTGAAAACAGCCAGGGAGA	Reverse primer for *nspS* cloning for mutagenesis
pNspS-F	TGATGGCGACGATGGAGAAC	Forward primer for amplifying the *nspS* promoter region
pNspS-R	GAACGACATCATGCAATCGC	Reverse primer for amplifying the *nspS* promoter region

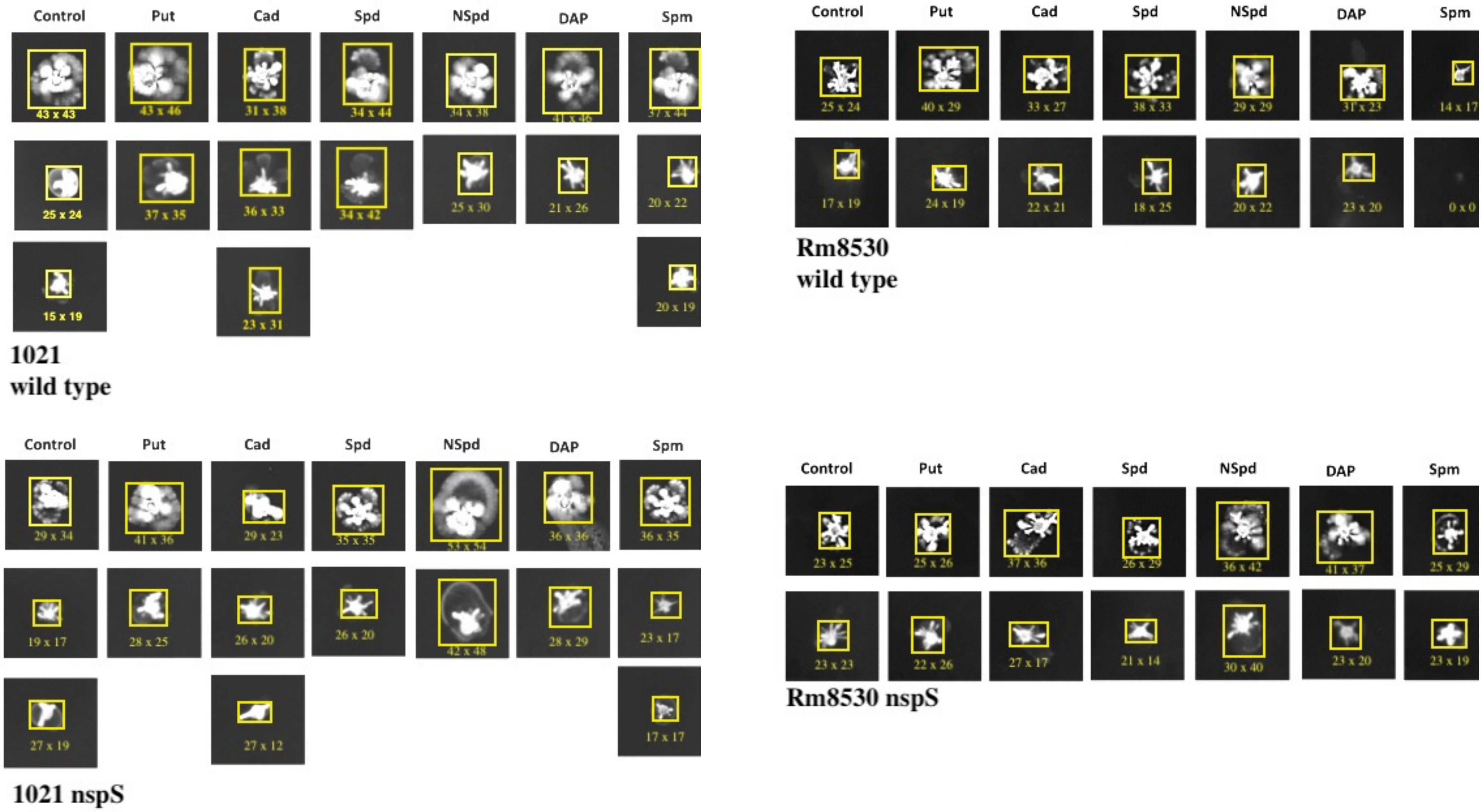

Control
Put
Cad
Spd
NSpd
DAP
Spm
43 x 43
43 x 46
31 x 38
34 x 44
34 x 38
25 x 24
37 x 35
36 x 33
34 x 42
25 x 30
21 x 26
20 x 22
15 x 19
23 x 31
20 x 19
1021
wild type
25 x 24
40 x 29
33 x 27
38 x 33
29 x 29
31 x 23
14 x 17
17 x 19
24 x 19
22 x 21
18 x 25
20 x 22
23 x 20
0 x 0
Rm8530
wild type
29 x 34
41 x 36
29 x 23
35 x 35
36 x 36
36 x 35
19 x 17
28 x 25
26 x 20
26 x 20
42 x 48
28 x 29
23 x 17
27 x 19
27 x 12
17 x 17
1021 nspS
23 x 25
25 x 26
37 x 36
26 x 29
36 x 42
41 x 37
25 x 29
23 x 23
22 x 26
27 x 17
21 x 14
30 x 40
23 x 20
23 x 19
Rm8530 nspS

Fig. S1

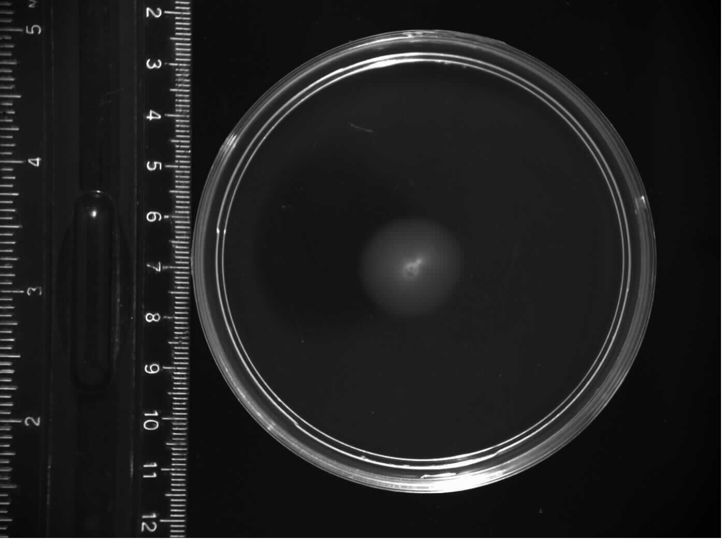
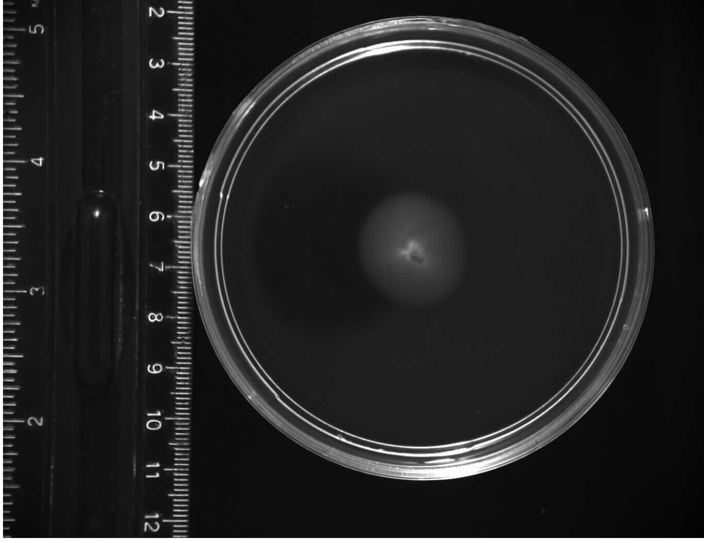

1021 wild type

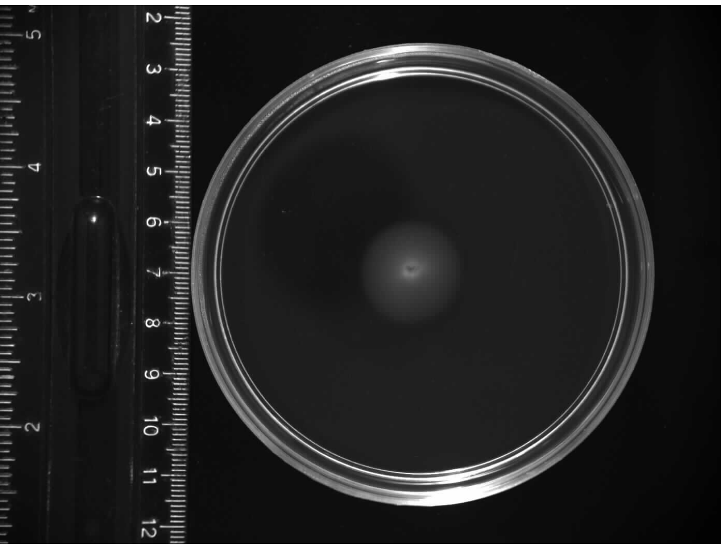
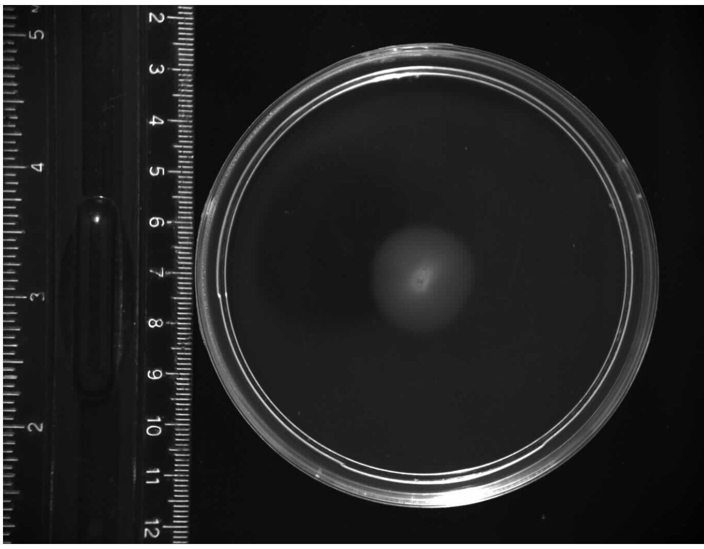

1021 nspS

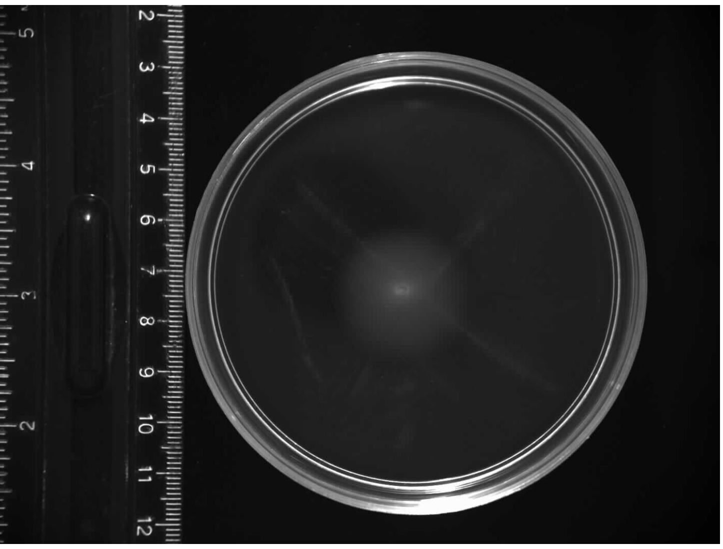
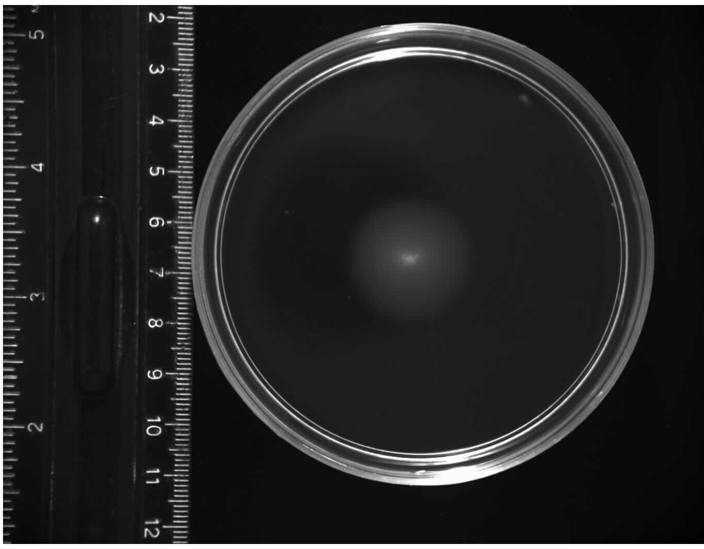

Rm8530 wild type

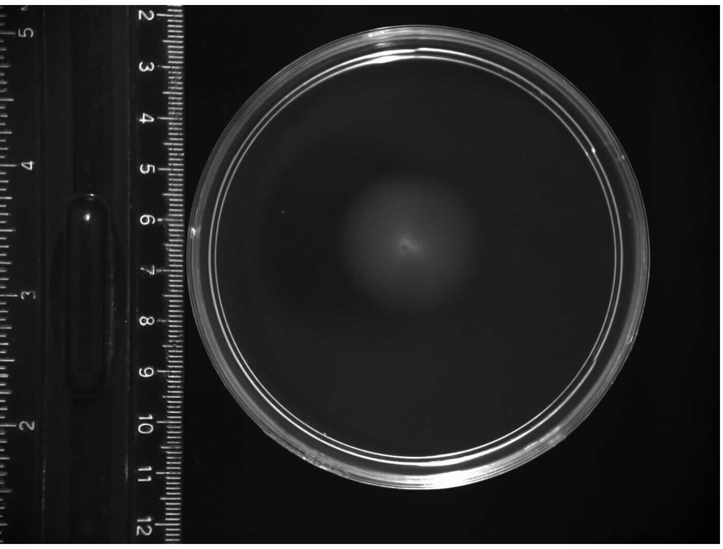
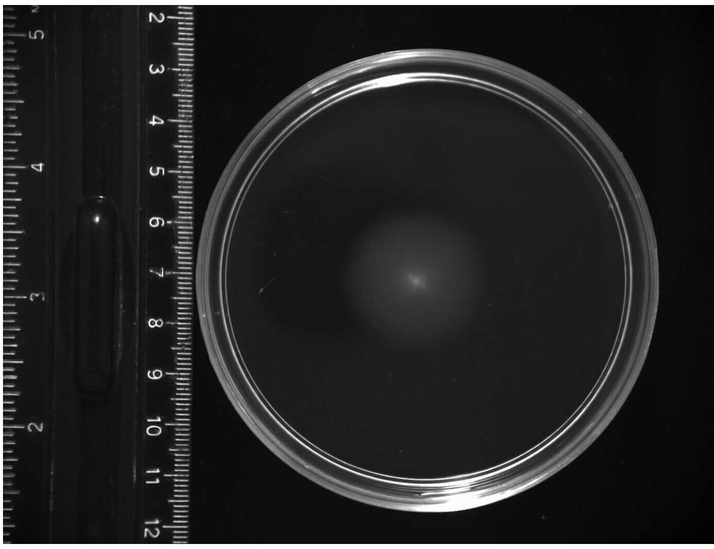

Rm8530 nspS

Fig. S2

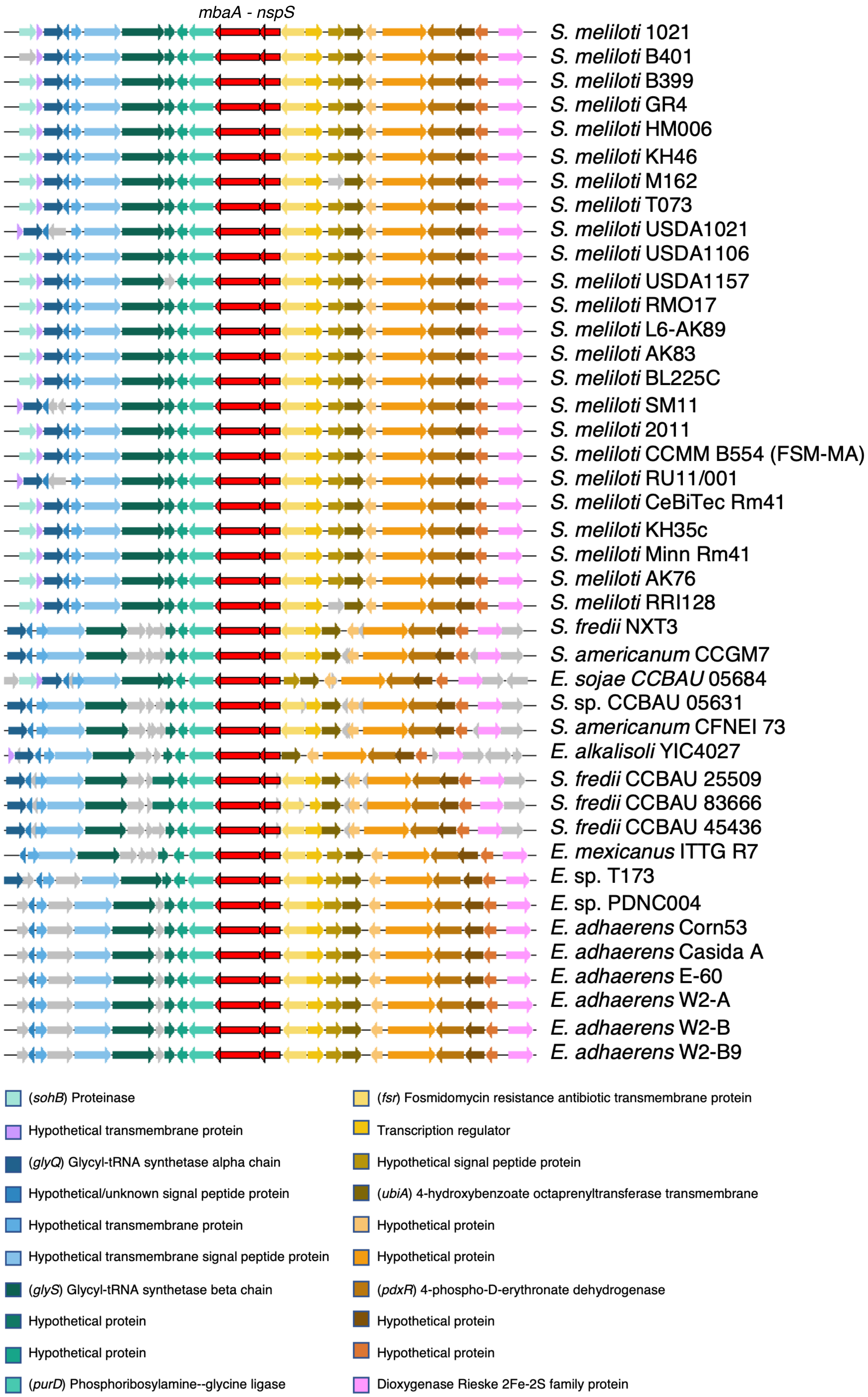
mbaA - nspS
S. meliloti 1021
S. meliloti B401
S. meliloti B399
S. meliloti GR4
S. meliloti HM006
S. meliloti KH46
S. meliloti M162
S. meliloti T073
S. meliloti USDA1021
S. meliloti USDA1106
S. meliloti USDA1157
S. meliloti RMO17
S. meliloti L6-AK89
S. meliloti AK83
S. meliloti BL225C
S. meliloti SM11
S. meliloti 2011
S. meliloti CCMM B554 (FSM-MA)
S. meliloti RU11/001
S. meliloti CeBiTec Rm41
S. meliloti KH35c
S. meliloti Minn Rm41
S. meliloti AK76
S. meliloti RRI128
S. fredii NXT3
S. americanum CCGM7
E. sojae CCBAU 05684
S. sp. CCBAU 05631
S. americanum CFNEI 73
E. alkalisoli YIC4027
S. fredii CCBAU 25509
S. fredii CCBAU 83666
S. fredii CCBAU 45436
E. mexicanus ITTG R7
E. sp. T173
E. sp. PDNC004
E. adhaerens Corn53
E. adhaerens Casida A
E. adhaerens E-60
E. adhaerens W2-A
E. adhaerens W2-B
E. adhaerens W2-B9
(sohB) Proteinase
Hypothetical transmembrane protein
(glyQ) Glycyl-tRNA synthetase alpha chain
Hypothetical/unknown signal peptide protein
Hypothetical transmembrane protein
Hypothetical transmembrane signal peptide protein
(glyS) Glycyl-tRNA synthetase beta chain
Hypothetical protein
Hypothetical protein
(purD) Phosphoribosylamine--glycine ligase
(fsr) Fosmidomycin resistance antibiotic transmembrane protein
Transcription regulator
Hypothetical signal peptide protein
(ubiA) 4-hydroxybenzoate octaprenyltransferase transmembrane
Hypothetical protein
Hypothetical protein
(pdxR) 4-phospho-D-erythronate dehydrogenase
Hypothetical protein
Hypothetical protein
Dioxygenase Rieske 2Fe-2S family protein

Fig. S3

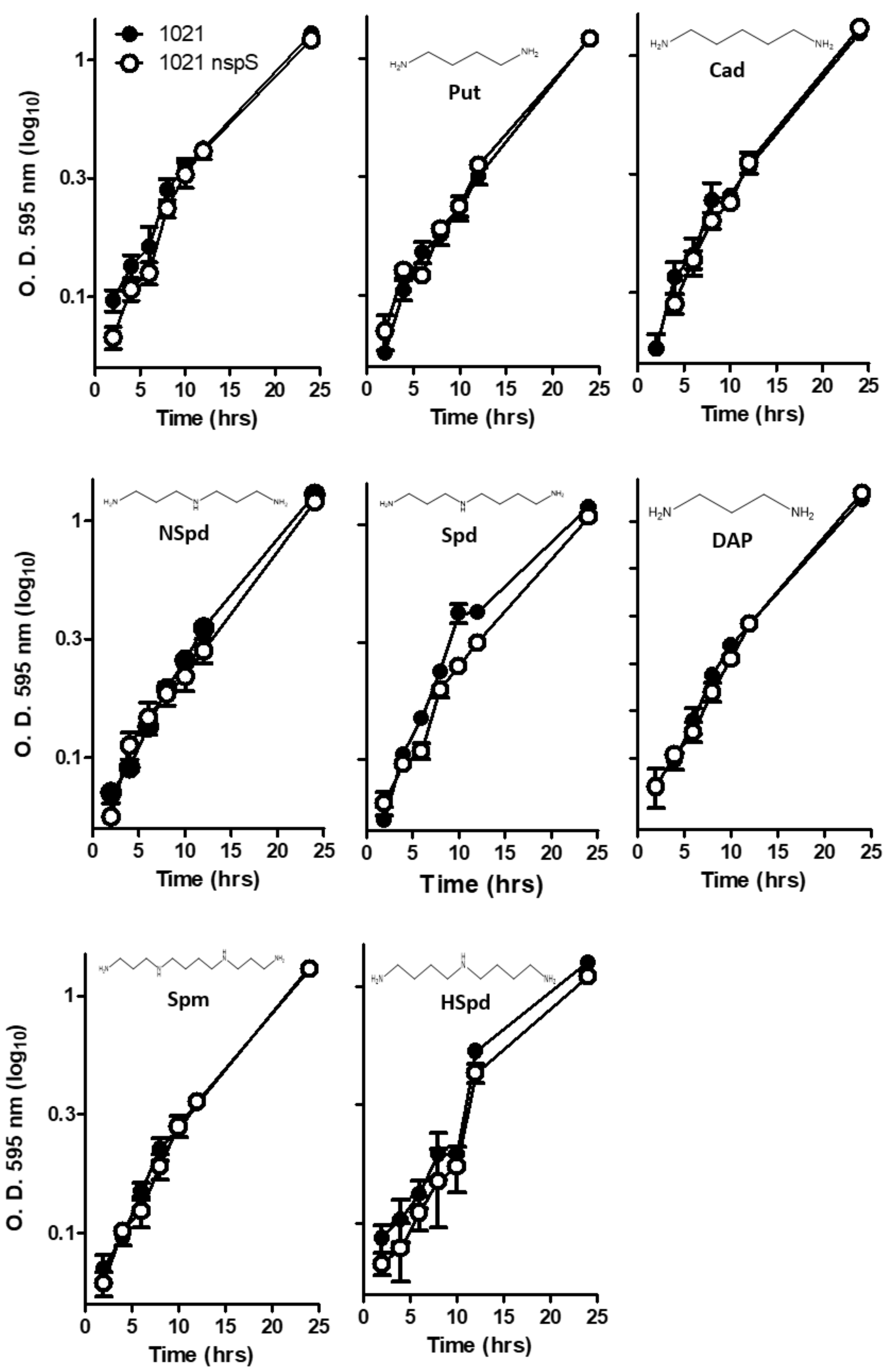
1021
1021 nspS
Put
Cad
NSpd
Spd
DAP
Spm
HSpd
O. D. 595 nm (log10)
Time (hrs)

Fig. S4

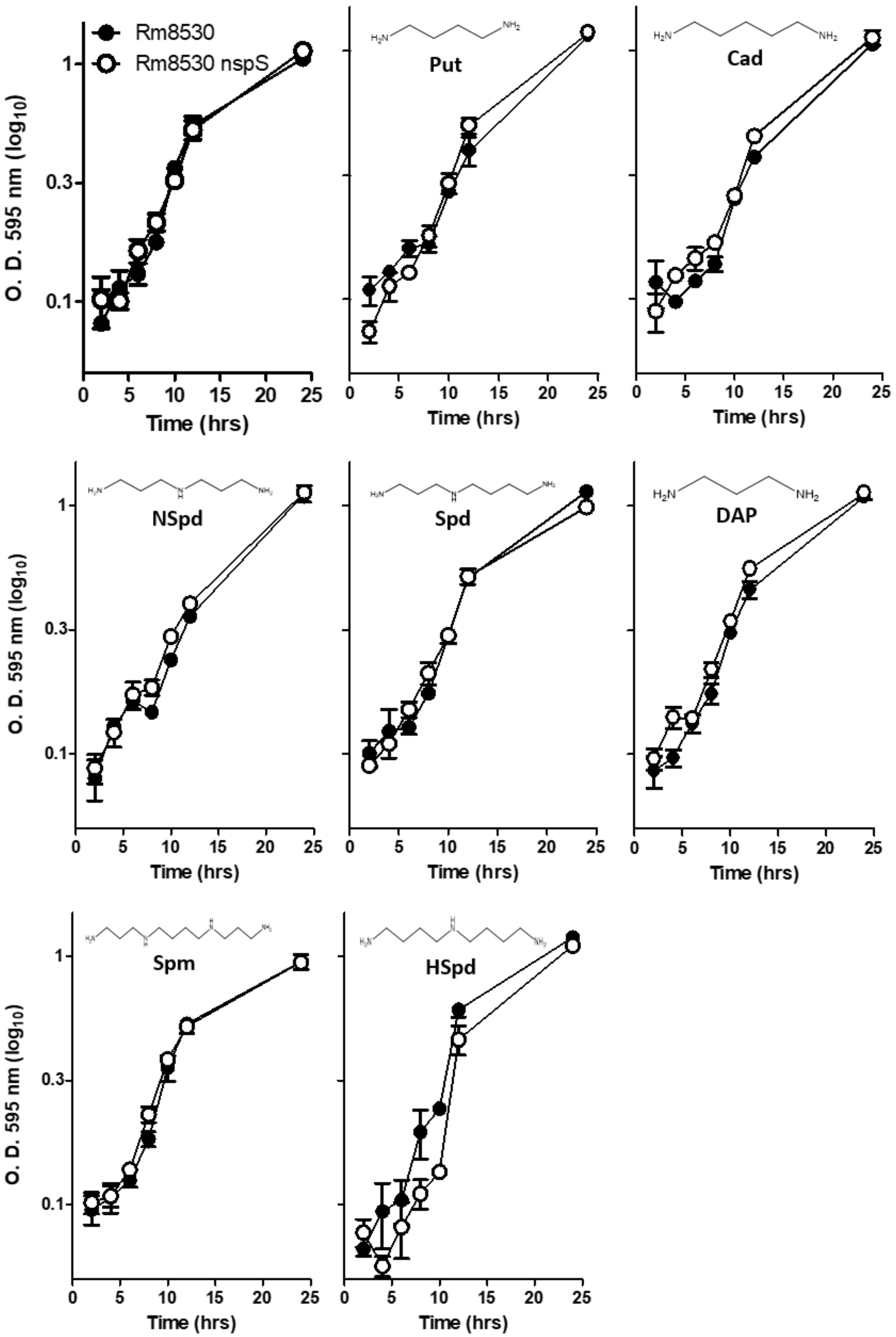
Rm8530
Rm8530 nspS
O. D. 595 nm (log10)
Time (hrs)
Put
Cad
NSpd
Spd
DAP
Spm
HSpd
H2N
NH2
1
0.3
0.1
0
5
10
15
20
25

Fig. S5
